# Trends in Gestational Weight Gain and Prepregnancy Obesity in South Carolina, 2015–2021

**DOI:** 10.5888/pcd21.240137

**Published:** 2024-12-12

**Authors:** Sarah E. Simpson, Angela M. Malek, Chun-Che Wen, Brian Neelon, Dulaney A. Wilson, Julio Mateus, John Pearce, Kalyan J. Chundru, Jeffrey E. Korte, Hermes Florez, Mallory Alkis, Matt Finneran, Kelly J. Hunt

**Affiliations:** 1 Department of Public Health Sciences, Medical University of South Carolina, Charleston; 2 Health Equity and Rural Outreach Innovation Center, Ralph H. Johnson Department of Veterans Affairs Medical Center, Charleston; 3 Department of Obstetrics and Gynecology, Maternal–Fetal Medicine Division, Atrium Health, Charlotte, North Carolina; 4 Department of Obstetrics & Gynecology, Medical University of South Carolina, Charleston

## Abstract

**Introduction:**

We examined trends in prepregnancy obesity and gestational weight gain, with a focus on racial and ethnic differences, before and during the COVID-19 pandemic in South Carolina.

**Methods:**

Hospital and emergency department discharge codes were linked to birth certificates. Prepregnancy obesity was defined as a body mass index (kg/m^2^) of 30 or higher. Gestational weight gain was defined as inadequate, adequate, or excessive based on the 2009 Institute of Medicine guidelines. A generalized linear model with a multinomial distribution and glogit link estimated the risk of inadequate weight gain and excessive weight gain with adequate weight gain as the reference group. The generalized linear model with a modified Poisson distribution and log link estimated prepregnancy obesity risk with nonobese as the reference group.

**Results:**

Our study included 306,344 full-term, singleton live births among 239,597 mothers from 2015 through 2021. The prevalence of inadequate weight gain increased across all racial and ethnic groups prepandemic (relative risk [RR] = 1.02; 95% CI, 1.01–1.02) and attenuated during the pandemic (RR = 0.99; 95% CI, 0.96–1.01). The prevalence of excessive weight gain was high and remained stable across all races and ethnicities before and during the pandemic. The prevalence of prepregnancy obesity increased across all racial and ethnic groups prepandemic; the prevalence after the start of the pandemic increased only among women of “other” races and ethnicities (RR = 1.12; 95% CI, 1.05–1.19) while attenuating among Hispanic, non-Hispanic Black, and non-Hispanic White women.

**Conclusion:**

The COVID-19 pandemic did not alter trends of gestational weight gain; however, it did have a small effect on trends in prepregnancy obesity, with differential effects across racial and ethnic groups. The prevalence of prepregnancy obesity, inadequate weight gain, and excessive weight gain remains high among pregnant women in South Carolina. Obesity and weight gain are risk factors for many adverse maternal and infant pregnancy outcomes. Their high prevalence indicates the importance of developing effective weight management programs for women of childbearing age and pregnant women.

**Summary**

**What is already known on this topic?**

The prevalence of prepregnancy obesity, inadequate weight gain, and excessive weight gain is high among pregnant women and varies by race and ethnicity. However, whether the COVID-19 pandemic (eg, food shortages, isolation due to lockdown measures) had a significant long-term effect on weight gain in this population is unclear.

**What is added by this report?**

The COVID-19 pandemic did not alter trends of gestational weight gain. It did, however, have a small effect on trends in prepregnancy obesity, with differential effects across racial and ethnic groups.

**What are the implications for public health practice?**

Prepregnancy obesity and gestational weight gain are public health issues that can lead to the development of adverse maternal and infant pregnancy outcomes, warranting effective public health interventions.

## Introduction

Over the past 40 years, obesity and weight gain have increased rapidly in the US, particularly among children, adolescents, and young adults. However, the literature is lacking assessment of how obesity and weight gain have changed over time among women of childbearing age. The Centers for Disease Control and Prevention’s (CDC’s) Pregnancy Risk Assessment Monitoring System and the National Vital Statistics System reported the prevalence of adequate weight gain during pregnancy as 32.1% during 2012 and 2013 (1). During the same period, the prevalence of inadequate weight gain during pregnancy was 20.4%, and the prevalence of excessive weight gain was 47.5%. Stratified by prepregnancy body mass index (BMI) (kg/m^2^) category, underweight women (32.2%) were more likely to gain inadequate weight during pregnancy, whereas 61.6% of overweight and 55.8% of obese women were more likely to gain excessive weight than women of normal weight (1).

CDC’s National Vital Statistics System reported that 27.2% of women were overweight before pregnancy and 30% had obesity in 2020. Among women who had obesity, 16.1% were classified as class I obese (BMI 30.0 to 34.9), 8.1% as class II obese (BMI 35.0 to 39.9), and 5.9% as class III obese (BMI ≥40.0) (2). Additionally, the prevalence of obesity was significantly higher among non-Hispanic Black women (40.3%) compared with non-Hispanic White (27.4%) and Hispanic women (33.6%) (2).

Prepregnancy obesity and gestational weight gain are associated with many adverse infant outcomes (low birthweight, preterm birth, large size for gestational age, admission to neonatal intensive care unit, macrosomia, childhood obesity, infant mortality) and poor maternal outcomes (cesarean delivery, gestational hypertension, preeclampsia) (3–7).

Although the association between prepregnancy obesity, gestational weight gain, and adverse maternal and infant outcomes has been established, few studies have focused on how the prevalence of these conditions has changed over time, especially during the COVID-19 pandemic. The pandemic has affected not only the health care system and subsequent health outcomes but also people’s physical activity and eating behaviors because of social distancing measures (both self-imposed and mandated) and disruptions in the US food supply chain. Initial studies on the pandemic’s effect on obesity and weight gain differ by whether the increase was significant (8–15). Our objective was to examine trends in prepregnancy obesity and gestational weight gain with a focus on racial and ethnic differences and associated sociodemographic and clinical factors before and during the COVID-19 pandemic in South Carolina, from January 2015 through December 2021.

## Methods

### Study design and population

Our sample population was South Carolina resident mothers who delivered live singleton births from January 2015 through December 2021. Because gestational weight gain is affected by preterm birth, we limited the population to full-term (37 weeks) deliveries. The South Carolina Department of Health and Environmental Control provided information from birth certificates. Data from birth certificates were linked to maternal inpatient hospital discharge records and emergency department (ED) visit records by the South Carolina Revenue and Fiscal Affairs office. Beginning in 2012, that office also provided data at least 3 years before each delivery on maternal inpatient discharges and ED visits to identify pre-existing health conditions. Database linkages were based on an algorithm created by the South Carolina Revenue and Fiscal Affairs office that used personal identifying information. The institutional review board of the Medical University of South Carolina approved our study as exempt research.

### Variable definition

Maternal race and ethnicity were categorized as Hispanic, non-Hispanic Black, non-Hispanic White, or “other” race or ethnicity based on what was commonly reported on birth certificate and inpatient and ED visit records. However, a mother was classified as Hispanic if she identified as Hispanic 3 or more times in the dataset. The “other” race or ethnicity group included women who self-identified as Asian, American Indian/Alaska Native, Native Hawaiian/Other Pacific Islander or for whom race/ethnicity was missing. Birth certificates reported education (categorized as less than high school graduate, high school diploma or General Educational Development [GED], some college, or undergraduate or associate degree or more); residence (rural vs urban); receipt of Special Supplemental Nutrition Program for Women, Infants, and Children (WIC) benefits during pregnancy; smoking during pregnancy or prepregnancy (smoker vs nonsmoker); and maternal prepregnancy weight and height. Women were classified as underweight (BMI 14.0–18.4), normal (BMI 18.5–24.9), overweight (BMI 25.0–29.9), or obese (BMI ≥30.0). For our analysis, the outcome of prepregnancy obesity was defined as obese versus nonobese. Firstborn was defined as the first live or stillborn birth from 2015 through 2021 of a mother without a history of a previous live birth or stillbirth on the birth certificate. Medicaid status was defined as being Medicaid eligible within 2 months of giving birth based on the statewide Medicaid eligibility file. Gestational weight gain was categorized as adequate, inadequate, or excessive based on the mother’s prepregnancy BMI, according to the 2009 Institute of Medicine guidelines (16). These guidelines state how much weight women with singleton pregnancies should gain during pregnancy based on the mother’s prepregnancy weight status: underweight, 28 to 40 lb; normal weight, 25 to 35 lb; overweight, 15 to 25 lb; and obese, 11 to 20 lb.

### Statistical analysis

We used the χ^2^ test in preliminary statistical analyses to examine bivariate associations between sociodemographic, lifestyle, and clinical factors and outcomes of interest by maternal racial and ethnic group. A generalized linear model with a modified Poisson distribution and log link was used to estimate the risk of prepregnancy obesity, with nonobese as the reference group. A second generalized linear model with a multinomial distribution and glogit link was used to estimate the risk of inadequate or excessive weight gain with adequate weight gain as the reference group. Modified Poisson models were used to express estimates as risk ratios (RRs) because log–binomial models can have convergence issues as the model’s complexity increases (17,18). Additionally, the point estimates of the modified Poisson model are proven to be unbiased when the link function is misspecified or the response rate is low (18). Generalized estimating equations with an exchangeable working correlation were used to account for mothers who had multiple pregnancies. To assess trends over time, a predetermined change point at the first quarter of 2020 (ie, March 2020), defining the start of the COVID-19 pandemic, was included in the models. No sensitivity analyses were conducted to assess robustness of results. Interaction terms were included to assess the association between racial and ethnic groups and trends over time. Covariates included in the models were identified a priori. For prepregnancy obesity, we ran an unadjusted model with the main effects of time before the change point, time after the change point, and race and ethnicity as well as interaction terms between time (before and after the change point) and race and ethnicity. For gestational weight gain, we ran an unadjusted model with the main effects of time before the change point, time after the change point and race and ethnicity. For both outcomes, models were adjusted for sociodemographic factors (age, education, rural residence, Medicaid, WIC receipt during pregnancy) and lifestyle and clinical factors (smoking during or prepregnancy, firstborn, prepregnancy BMI). 

We then plotted the prevalence of each outcome from 2015 to 2021 by using the unadjusted models of each outcome for the specified period with 95% CIs. *P* values of.05, and corresponding 95% CIs were used to determine significance. Analyses were conducted in SAS (SAS Institute), and figures were created in R (R Foundation) software.

## Results

### Study population

**Figure 1:**
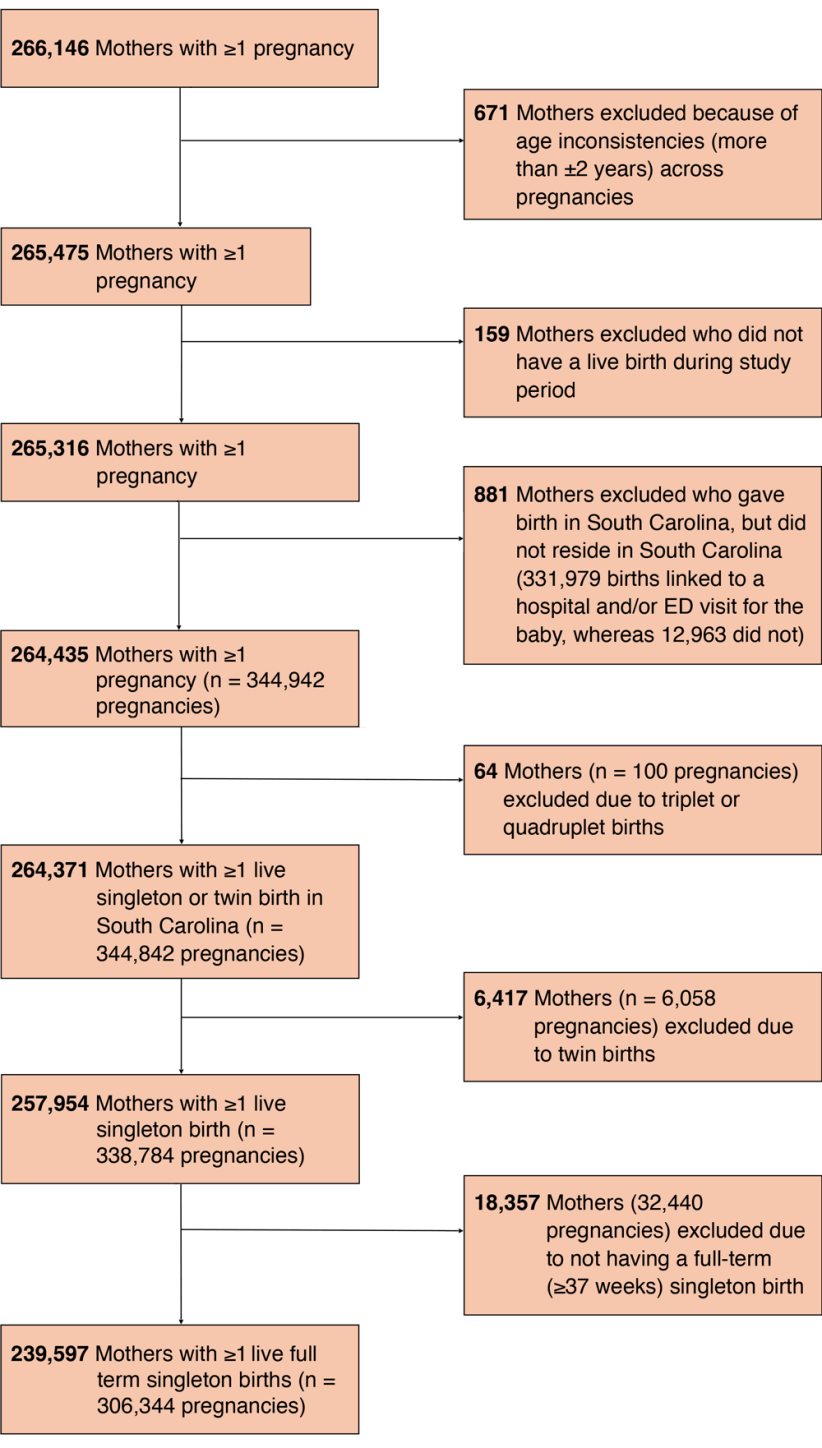
Flowchart of exclusion criteria for study sample, study of trends in gestational weight gain and prepregnancy obesity in South Carolina, 2015 through 2021.

Of 266,146 South Carolina mothers with at least 1 pregnancy from 2015 through 2021 (331,979 births), 671 (0.25%) were excluded because information on maternal age was inconsistent across multiple sources (defined as varying by more than ±2 years). We excluded 159 mothers (0.06%) who did not have a live birth during the study time frame, 881 (0.33%) who resided outside South Carolina, 64 (0.02%) who had a live birth of triplets or quadruplets during the study period, 6,417 (2.4%) who had a twin birth, and lastly, 18,357 (7.1%) who did not have a full-term (≥37 weeks) singleton birth. The final dataset consisted of 239,597 mothers with 1 or more live, full-term, singleton births (306,344 pregnancies) (Figure 1). Some sociodemographic, lifestyle, and clinical information was available for all mothers from linked inpatient hospital and ED visit data procedure and diagnostic code files.

Characteristics of the 306,344 pregnancies resulting in a live singleton birth varied by race and ethnicity (Table 1). From 2015 through 2021, 57.4% of pregnancies were among non-Hispanic White women, 30.2% were among non-Hispanic Black women, 7.6% were among Hispanic women, and 4.8% were among women of other racial or ethnic groups. Average (SD) age at delivery ranged from 29.1 (5.9) years among women of other races or ethnicities to 26.7 (5.7) years among non-Hispanic Black women. Among Hispanic women, approximately 42.9% had less than a high school education, compared with only 9.4% of non-Hispanic White women. Medicaid eligibility at delivery was 72.2% among non-Hispanic Black women, 70.4% among Hispanic women, 49.4% among women of other racial or ethnic groups, and 39.1% among non-Hispanic White women. WIC receipt during pregnancy was 61.8% among non-Hispanic Black women, 43.9% among Hispanic women, 31.5% among women of other racial or ethnic groups, and 27.5% among non-Hispanic White women. Maternal prepregnancy obesity ranged from 44.8% of pregnancies among non-Hispanic Black women to 22.2% of pregnancies among women of other racial or ethnic groups. Excessive weight gain during pregnancy ranged from 51.8% of pregnancies among non-Hispanic White women to 39.2% of pregnancies among Hispanic women. 

**Table 1:** Characteristics of 306,344 Pregnancies Resulting in a Live Full-Term (≥37 Weeks) Singleton Birth, South Carolina, 2015–2021^a^

Characteristic	Racial and or ethnic group			
	Non-Hispanic White (n = 175,991)	Non-Hispanic Black (n = 92,402)	Hispanic (n = 23,423)	Other (n = 14,708)
**Sociodemographic**				
**Age at delivery, mean (SD), y**	28.4 (5.5)	26.7 (5.7)	28.2 (6.1)	29.1 (5.9)
**Education, %^b^**				
Less than high school education	9.4	13.3	42.9	17.0
High school diploma or GED	20.0	34.4	27.0	20.6
Some college	23.1	30.4	13.6	17.8
College or associates degree or more	47.5	22.0	16.5	44.6
**Rural residence, %**	29.9	36.0	28.1	23.3
**Medicaid eligibility at delivery, %**	39.1	72.2	70.4	49.4
**WIC receipt during pregnancy, %^a^**	27.5	61.8	43.9	31.5
**Lifestyle and clinical factors**				
**Smoking during or prepregnancy, %^a^**	14.8	8.5	2.0	4.7
**Firstborn, %^b^**	33.1	29.3	25.9	34.2
**Prepregnancy BMI (kg/m^2^), %^b^**				
Underweight (<18.5)	3.6	2.8	2.0	4.6
Normal (18.5–24.9)	44.2	27.6	36.5	46.7
Overweight (25.0–29.9)	25.1	24.8	32.4	26.6
Obese (≥30.0)^b^	27.1	44.8	29.1	22.2
**Gestational weight gain, %^b^^,^ c**				
Adequate	29.6	26.3	32.1	31.4
Inadequate	18.7	28.4	28.6	28.5
Excessive	51.8	45.3	39.2	40.2

Abbreviations: BMI, body mass index; GED, General Educational Development; WIC, Special Supplemental Nutrition Program for Women, Infants, and Children.

a Stratified by racial and ethnic group.

b Number of women with missing data values on outcomes and covariates: education, 844; smoking during or prepregnancy, 195; firstborn, 66; prepregnancy BMI, 3,696; WIC, 14; prepregnancy obesity, 3,696; gestational weight gain classification, 3,696.

c Adequate weight gain during pregnancy for women who were underweight was 50 to 62 lb; normal weight gain, 25 to 35 lb; overweight, 15 to 25 lb; and obese, 11 to 20 lbs. Inadequate weight gain was defined as gaining less than the recommended weight during pregnancy. Excessive weight gain was defined as gaining more than the recommended weight during pregnancy. In our study, 87,350 women gained adequate weight during pregnancy, 68,998 women gained inadequate weight, and 146,300 gained excessive weight.

### Gestational weight gain by race and ethnicity

In the assessment of unadjusted trends in gestational weight gain before and after the start of the COVID-19 pandemic, the interactions between time and race and ethnicity were not significant (*P* = .30 and .47, respectively), indicating that trends over time were similar across all racial and ethnic groups.

**Figure 2:**
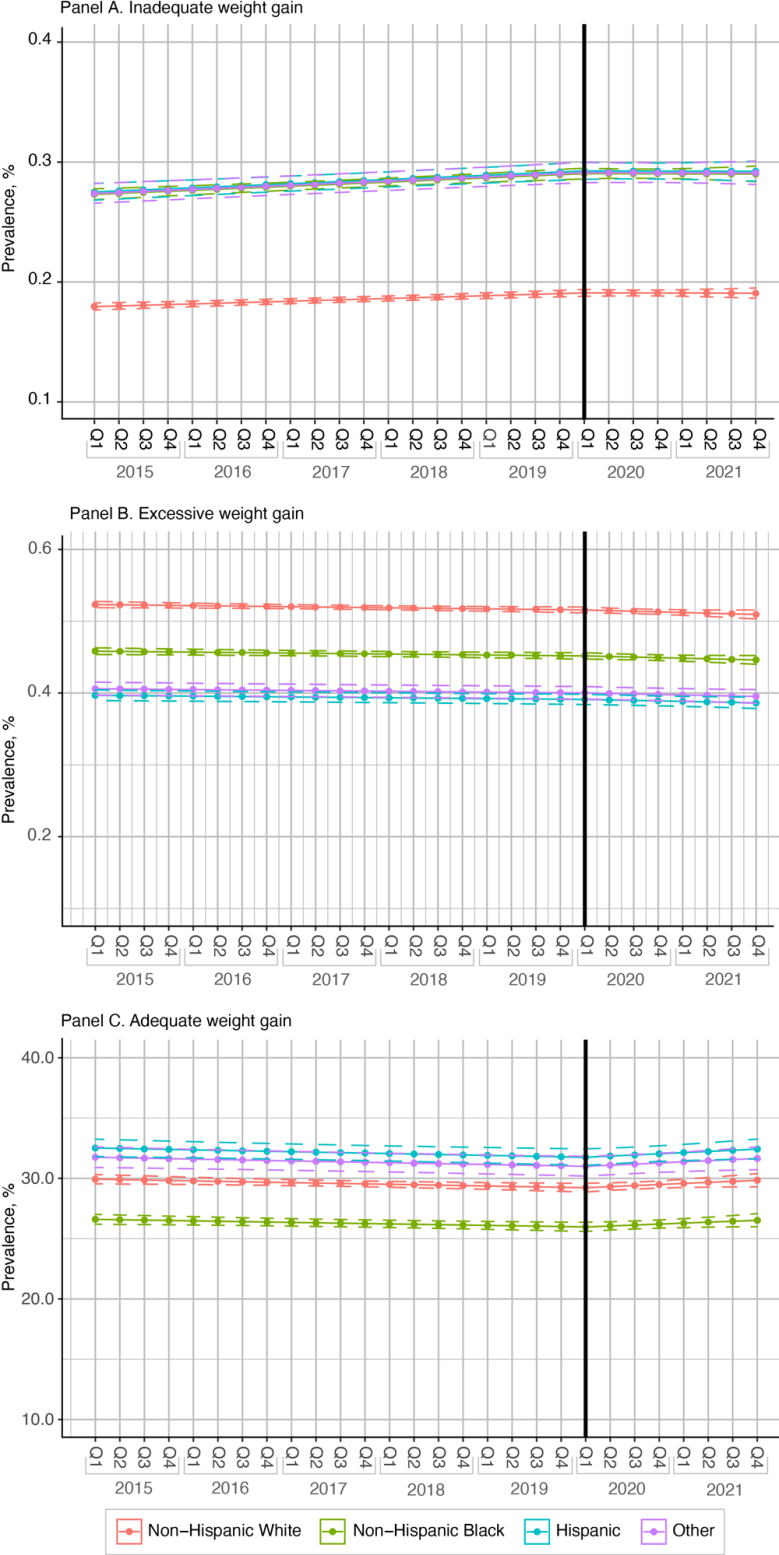
Prevalence of 3 categories of gestational weight gain among women with 1 or more full-term (≥37 weeks) singleton births in South Carolina, by race or ethnicity, from 2015 through 2021: inadequate weight gain (Panel A), excessive weight gain (Panel B), and adequate weight gain (Panel C). Thick black vertical line indicates the start of the COVID-19 pandemic. Dotted lines indicate 95% CIs. Other race or ethnicity includes women who self-identified as Asian, American Indian/Alaska Native, Native Hawaiian/Other Pacific Islander or those whose race/ethnicity was missing. Abbreviation: Q, quarter of year.

**Inadequate weight gain.** For non-Hispanic White women, the prevalence of inadequate weight gain in 2015, quarter 1 was 18.0%; in 2020, quarter 1, 19.1%; and in 2021, quarter 4, 19.1% (Figure 2, Panel A). Among non-Hispanic Black women, the prevalence in 2015, quarter 1 was 27.3%; in 2020, quarter 1, 29.0%; and in 2021, quarter 4, 29.0%. Among Hispanic women, the prevalence in 2015, quarter 1 was 27.5%; in 2020, quarter 1, 29.3%; and in 2021, quarter 4, 29.2%. The prevalence among women of other races or ethnicities in 2015, quarter 1 was 27.4%; in 2020, quarter 1, 29.1%; and in 2021, quarter 4, 29.1%.

In the unadjusted model assessing the main effect for race and ethnicity (Table 2, Model 1), the RR for inadequate weight gain relative to adequate weight gain for a 1-year increase in calendar time was 1.02 (95% CI, 1.01–1.02) before the pandemic (ie, change point) and 0.99 (95% CI, 0.96–1.01) after the pandemic (ie, after the change point). Across all racial and ethnic groups, non-Hispanic Black (RR = 1.71, 95% CI, 1.67–1.75), Hispanic (RR = 1.41; 95% CI, 1.36–1.46), and women of other racial and ethnic groups (RR = 1.44; 95% CI, 1.37–1.51) were more likely to gain inadequate relative to adequate weight during each pregnancy compared with non-Hispanic White women.

**Table 2:** Trends in Inadequate and Excessive Weight Gain Among Live Full Term (≥37 Weeks) Singleton Births, Unadjusted and Adjusted for Sociodemographic and Lifestyle and Clinical Factors, South Carolina, 2015–2021

Characteristic	Inadequate, relative risk (95% CI)^a^		Excessive, relative risk (95% CI)^a^	
	Model 1^b^	Model 2^c^	Model 1^b^	Model 2^c^
Time before change point (per year)^d^	1.02 (1.01–1.02)^e^	1.02 (1.01–1.03)^e^	1.00 (1.00–1.01)	1.00 (0.99–1.00)
Time after change point (per year)^d^	0.99 (0.96–1.01)	0.99 (0.97–1.02)	0.98 (0.96–1.00)	0.98 (0.96–1.00)
**Trend by sociodemographic characteristic**				
**Race or ethnicity**				
Non-Hispanic White	1 [Reference]	1 [Reference]	1 [Reference]	1 [Reference]
Non-Hispanic Black	1.71 (1.67–1.75)^e^	1.45 (1.42–1.49)^e^	0.99 (0.97–1.01)	0.85 (0.83–0.87)^e^
Hispanic	1.41 (1.36–1.46)^e^	1.17 (1.13–1.22)^e^	0.70 (0.67–0.72)^e^	0.67 (0.65–0.69)^e^
Other^f^	1.44 (1.37–1.51)^e^	1.42 (1.36–1.49)^e^	0.73 (0.70–0.76)^e^	0.76 (0.73–0.79)^e^
**Age at delivery (per year)**	—g	1.00 (0.995–0.996)^e^	—g	1.00 (0.994–0.998)^e^
**Education**				
Less than high school education	1 [Reference]	1 [Reference]	1 [Reference]	1 [Reference]
High school diploma or GED	—g	0.85 (0.82–0.88)^e^	—g	1.08 (1.05–1.12)^e^
Some college	—g	0.73 (0.70–0.76)^e^	—g	1.13 (1.10–1.17)^e^
College or associate degree or more	—g	0.62 (0.60–0.64)^e^	—g	1.08 (1.04–1.12)^e^
**Rural residence (vs urban)**	—g	1.07 (1.05–1.10)^e^	—g	1.01 (0.99–1.03)
**Medicaid eligibility at delivery (yes vs no)**	—g	1.11 (1.08–1.14)^e^	—g	1.01 (0.99–1.03)
**WIC receipt during pregnancy **(yes vs no)	—g	1.01 (0.99–1.04)	—g	1.05 (1.02–1.07)^e^
**Trends by lifestyle and clinical factors**				
Smoking during or prepregnancy (yes vs no)	—g	1.07 (1.03–1.10)^e^	—g	1.26 (1.22–1.30)^e^
Firstborn (yes vs no)	—g	0.91 (0.88–0.93)^e^	—g	1.31 (1.28–1.33)^e^
**Prepregnancy BMI (kg/m^2^)**				
Underweight (<18.5)	—g	1.02 (0.97–1.07)	—g	0.55 (0.52–0.58)^e^
Normal (18.5–24.9)	—g	1 [Reference]	—g	1 [Reference]
Overweight (25.0–29.9)	—g	0.79 (0.77–0.81)^e^	—g	2.26 (2.21–2.32)^e^
Obese (≥30.0)	—g	1.28 (1.25–1.32)^e^	—g	2.11 (2.06–2.15)^e^

Abbreviations: BMI, body mass index; GED, General Education Development; WIC, Special Supplemental Nutrition Program for Women, Infants, and Children.

a Relative risks represent the risk of inadequate and excessive weight gain for a 1-year increase in calendar time.

b Model 1: relative risks for time before and after change point (first quarter of 2020) for the main effect for race and ethnicity. The change point is a predetermined point at the first quarter of 2020 (ie, March 2020) defining the start of the COVID-19 pandemic.

c Model 2: relative risks for time before and after change point (first quarter of 2020) adjusted for sociodemographic characteristics and lifestyle and clinical factors. The change point is a predetermined point at the first quarter of 2020 (ie, March 2020) defining the start of the COVID-19 pandemic.

d Interaction *P* value for time before change point and race or ethnicity was.30. Interaction *P* value for time after change point and race or ethnicity was .47 in Model 1. The change point is a predetermined point at the first quarter of 2020 (ie, March 2020) defining the start of the COVID-19 pandemic.

e Significant at *P* <. 05.

f Includes women who self-identified as Asian, American Indian/Alaska Native, Native Hawaiian/Other Pacific Islander, or those whose race/ethnicity was missing or unknown.

g Indicates no relative risks were estimated for sociodemographic characteristics and lifestyle and clinical factors.

In the fully adjusted model (Table 2, Model 2), the RR of inadequate weight gain relative to adequate weight gain for a 1-year increase in calendar time before the pandemic (ie, change point) was 1.02 (95% CI, 1.01–1.03) and 0.99 (95% CI, 0.97–1.02) after the start of the pandemic (ie, after the change point). Age, higher maternal education, Medicaid eligibility, rural residence, smoking during or prepregnancy, having a firstborn, and having obesity or being overweight prepregnancy were associated with inadequate weight gain during pregnancy.

**Excessive weight gain.** Among non-Hispanic White women, the prevalence of excessive weight gain for pregnancies in 2015, quarter 1, was 52.3%; in 2020, quarter 1, 51.6%; and in 2021, quarter 4, 50.9% (Figure 2, Panel B). Among non-Hispanic Black women, the prevalence in 2015, quarter 1 was 45.8%; in 2020, quarter 1, 45.2%; and in 2021, quarter 4, 44.6%. Among Hispanic women, the prevalence in 2015, quarter 1 was 39.7%; in 2020, quarter 1, 39.1%; and in 2021, quarter 4, 38.6%. Among women of other races or ethnicities, the prevalence in 2015, quarter 1 was 40.6%; in 2020, quarter 1, 40.0%; and in 2021, quarter 4, 39.5%.

In the unadjusted model assessing the main effect of race and ethnicity (Table 2, Model 1), the RR for excessive weight gain relative to adequate weight gain for a 1-year increase in calendar time was 1.00 (95% CI, 1.00–1.01) before the pandemic (ie, before the change point) and 0.98 (95% CI, 0.96–1.00) after the start of pandemic (ie, after the change point). Across racial and ethnic groups, non-Hispanic Black women (RR = 0.99, 95% CI, 0.97–1.01) had similar risk during each pregnancy of excessive weight gain, whereas Hispanic women (RR = 0.70; 95% CI, 0.67–0.72) and women of other racial and ethnic groups (RR = 0.73; 95% CI, 0.70–0.76) were less likely to gain excessive weight compared with non-Hispanic White women.

In the fully adjusted model (Table 2, Model 2), the risk of excessive weight gain relative to adequate weight gain for a 1-year increase in calendar time before the pandemic (ie, before the change point) was 1.00 (95% CI, 0.99–1.00) and 0.98 (95% CI, 0.96–1.00) after the start of the pandemic (ie, after the change point). Age, higher maternal education, WIC receipt during pregnancy, smoking during or prepregnancy, having a firstborn, and having obesity or being overweight before pregnancy were associated with increased likelihood of excessive weight gain during pregnancy.

**Adequate weight gain.** Across all groups, the prevalence of adequate weight gain decreased before the pandemic and rose after the pandemic (Figure 2, Panel C). The prevalence of adequate weight gain among non-Hispanic White women in 2015, quarter 1, was 30.0%; in 2020, quarter 1, 29.2%; and in 2021, quarter 4, 29.8%. Among non-Hispanic Black women, the prevalence in 2015, quarter 1 was 26.6%; in 2020, quarter 1, 26.0%; and in 2021, quarter 4, 26.5%. Among Hispanic women, the prevalence in 2015, quarter 1 was 32.5%; in 2020, quarter 1, 31.8%; and 2021, quarter 4, 32.4%. Among women of other races or ethnicities, the prevalence in 2015, quarter 1 was 31.8%; in 2020, quarter 1, 31.0%; and in 2021, quarter 4, 31.7%.

### Obesity

**Figure 3:**
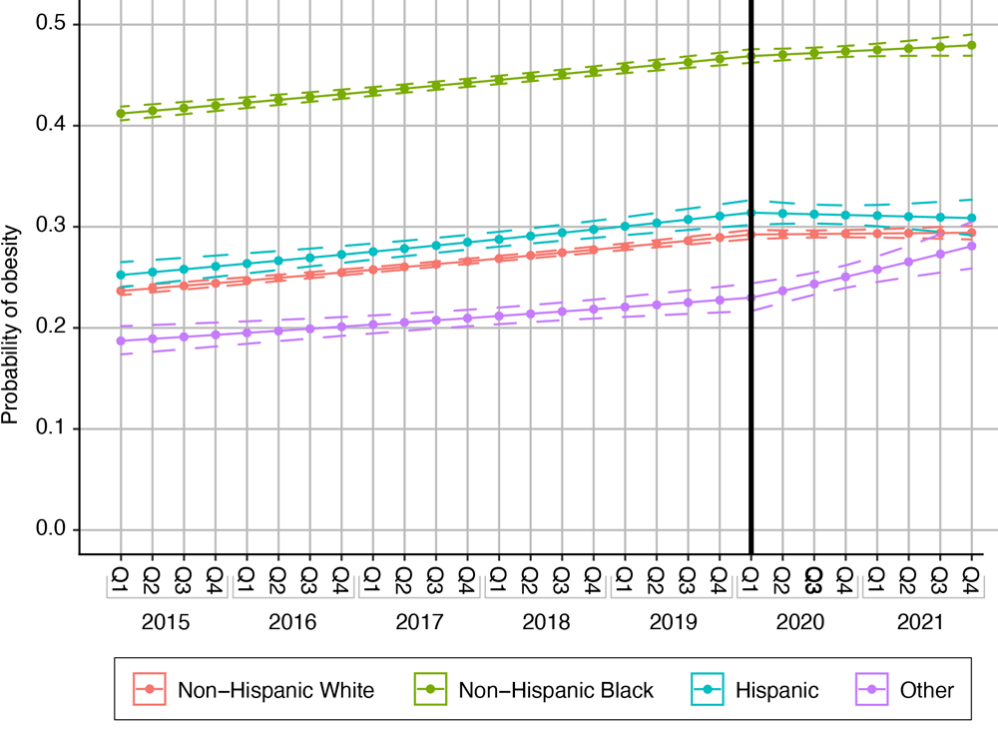
Prevalence of prepregnancy obesity among women with 1 or more full term (≥37 weeks) singleton births in South Carolina, by race and ethnicity, from 2015 through 2021. The change point was the start of the COVID-19 pandemic, quarter (Q) 1, the first quarter of 2020. Dotted lines indicate 95% CIs.

The prevalence of prepregnancy obesity was 23.7% in 2015 quarter 1, 29.2% in 2020 quarter 1, and 29.4% in 2021 quarter 4 for non-Hispanic White women (Figure 3). For non-Hispanic Black women, the prevalence of prepregnancy obesity was 41.2% in 2015, quarter 1 and increased to 47.0% in 2020, quarter 1, then further increased to 48.0% in 2021, quarter 4. For Hispanic women, prepregnancy obesity increased from 25.2% to 31.4% between 2015, quarter 1 and 2020, quarter 1, and then decreased slightly to 31.0 % in 2021, quarter 1. Among women of other racial and ethnic groups, the prevalence of prepregnancy obesity in 2015, quarter 1 was 18.7% then increased to 23% in 2020, quarter 1 and further increased to 28.1% in 2021, quarter 4.

RRs of prepregnancy obesity, unadjusted and adjusted for sociodemographic and lifestyle and clinical factors, varied by racial and ethnic groups before and after the change point (start of the pandemic, 2020, quarter 1) (Table 3). Temporal trends differed by racial or ethnic group before (*P* =. 002) and after (*P* =. 03) the pandemic. In the model assessing the main effect of race and ethnicity (Table 3, Model 1), the RR of prepregnancy obesity among non-Hispanic White women for a 1-year increase in calendar time before the pandemic was 1.04 (95% CI, 1.04–1.05); among non-Hispanic Black women, 1.03 (95% CI, 1.02–1.03); among Hispanic women, 1.04 (95% CI, 1.03–1.06); and among women of other races or ethnicities, 1.04 (95% CI, 1.02–1.07). After the pandemic, the risk of prepregnancy obesity for a 1-year increase in calendar time attenuated among non-Hispanic White (RR = 1.01, 95% CI, 0.99–1.02), non-Hispanic Black (RR = 1.01, 95% CI: 1.00–1.03) and Hispanic women (RR = 0.99, 95% CI, 0.95–1.04). However, among women of other racial and ethnic groups, the risk of prepregnancy obesity for a 1-year increase in calendar time increased significantly after the pandemic (RR = 1.12, 95% CI, 1.05–1.19).

**Table 3:** Trends in Prepregnancy Obesity Among Live, Full Term (≥37 Weeks) Singleton Births, Unadjusted and Adjusted for Sociodemographic and Lifestyle and Clinical Factors, South Carolina, 2015–2021

Characteristic	Prepregnancy obesity, relative risk (95% CI)^a^	
	Model 1^b^	Model 2^c^
**Time before change point (per year)^d^**		
**Race or ethnicity**		
Non-Hispanic White	1.04 (1.04–1.05)^e^	1.04 (1.04–1.05)^e^
Non-Hispanic Black	1.03 (1.02–1.03)^e^	1.03 (1.02–1.03)^e^
Hispanic	1.04 (1.03–1.06)^e^	1.06 (1.04–1.07)^e^
Other^f^	1.04 (1.02–1.07)^e^	1.05 (1.03–1.07)^e^
**Time after change point (per year)^a^**		
**Race or ethnicity**		
Non-Hispanic White	1.01 (0.99–1.02)	1.01 (0.99–1.02)
Non-Hispanic Black	1.01 (1.00–1.03)	1.01 (1.00–1.03)
Hispanic	0.99 (0.95–1.04)	1.00 (0.96–1.04)
Other^f^	1.12 (1.05–1.19)^e^	1.13 (1.06–1.20)^e^
**Trend by sociodemographic characteristic**		
**Age at delivery (per year)**	**—** g	1.02 (1.02–1.03)^e^
**Education**		
Less than high school education	**—** g	1 [Reference]
High school diploma or GED	**—** g	1.05 (1.03–1.07)^e^
Some college	**—** g	1.09 (1.07–1.11)^e^
College or associates degree or more	**—** g	0.84 (0.82–0.85)^e^
**Rural residence (vs urban)**	**—** g	1.11 (1.10–1.13)^e^
**Medicaid eligibility at delivery (yes vs no)**	**—** g	1.11 (1.09–1.12)^e^
**WIC receipt during pregnancy (yes vs no)**	**—** g	1.21 (1.19–1.22)^e^
**Trends by lifestyle and clinical characteristic**		
Smoking during or prepregnancy (yes vs no)	**—** g	0.94 (0.92–0.96)^e^
Firstborn (yes vs no)	**—** g	0.89 (0.88–0.90)^e^

Abbreviations: BMI, body mass index; GED, General Educational Development; WIC, Supplemental Nutrition Program for Women, Infants, and Children.

a Relative risks represent the risk of prepregnancy obesity for a 1-year increase in calendar time.

b Model 1: relative risks for the interaction of time before and after the change point (first quarter of 2020) and the main effect for race and ethnicity. The change point is a predetermined point at the first quarter of 2020 (ie, March 2020) defining the start of the COVID-19 pandemic.

c Model 2: relative risks for the interaction of time before and after the change point (first quarter of 2020) adjusted for sociodemographic characteristics and lifestyle and clinical factors. The change point is a predetermined point at the first quarter of 2020 (ie, March 2020) defining the start of the COVID-19 pandemic.

d Interaction *P* value for time before the change point and race or ethnicity was <.001. Interaction *P* value for time after change point and race and ethnicity was. 03 in Model 1. The change point is a predetermined point at the first quarter of 2020 (ie, March 2020) defining the start of the COVID-19 pandemic.

e Significant at *P* <.05.

f Includes women who self-identified as Asian, American Indian/Alaska Native, Native Hawaiian/Other Pacific Islander, or those whose race/ethnicity was missing.

g Indicates no relative risks were estimated for sociodemographic characteristics and lifestyle and clinical factors.

In the fully adjusted model (Table 3, Model 2), RRs of prepregnancy obesity for a 1-year increase in calendar time before and after the pandemic for racial and ethnic groups were similar to their unadjusted values after adjusting for sociodemographic, lifestyle and clinical factors. Age, higher maternal education, rural residence, Medicaid eligibility at delivery, and WIC eligibility during pregnancy were significantly associated with an elevated risk of prepregnancy obesity. 

## Discussion

The objective of our study was to assess trends in gestational weight gain and prepregnancy obesity before and after March 2020 in South Carolina because we believed trends would be significantly affected by the COVID-19 pandemic in its early stage. Our principal findings showed the relative prevalence of prepregnancy obesity increased 3% to 4% per year across all racial and ethnic groups before the pandemic; however, the level stabilized after the pandemic for non-Hispanic White and Hispanic women, while increasing rapidly among non-Hispanic Black women and women of other racial and ethnic groups. The prevalence of inadequate weight gain increased 1% to 2% across all racial and ethnic groups before the pandemic and then stabilized afterwards. The prevalence of inadequate weight gain was significantly higher among non-Hispanic Black women, Hispanic women, and women of other racial and ethnic groups across the whole study period compared with non-Hispanic White women. In contrast, the prevalence of excessive weight gain was high across all racial and ethnic groups and remained stable before the pandemic, while decreasing slightly after the pandemic.

Literature on the COVID-19 pandemic’s effect on body weight, prepregnancy BMI, and gestational weight gain among women of reproductive age (both teens and adults) remains sparse, although preliminary studies have begun to emerge. Two US studies reported a significant increase (0.06 kg and 0.46 kg) in gestational weight gain during the COVID-19 pandemic (19,20). Additionally, among women who were obese before pregnancy, gestational weight gain increased 0.17 kg during the pandemic (19). However, a Washington State study found a nonsignificant decrease in gestational weight gain (11.2 ±4.3 kg vs 10.6 ±5.4 kg) between women who delivered before and during the pandemic (21).

Though studies assessing the effect of the COVID-19 pandemic on prepregnancy weight and gestational weight gain among pregnant women are limited, several studies have been published on the effect of the pandemic on body weight, weight gain, and dietary and lifestyle behaviors among the overall adult population in the US and worldwide. In general, the pandemic appears to have had mixed effects on eating and lifestyle behaviors, because the prevalence of weight gain and mean increase in body weight and BMI varied between studies, with some people gaining weight and others losing weight. Most studies found that weight gain was due to physical inactivity, sedentary behaviors (eg, increased screen time), unhealthy eating habits (eg, increased consumption of highly processed food, increased number of meals, snacking, alcohol consumption), reduced sleep, emotional eating, stress, depression, and anxiety (8–15). People who were overweight and obese before the pandemic were more likely to gain weight during the pandemic (12–14).

Although the aforementioned studies showed that the pandemic affected body weight, weight gain, and eating and lifestyle behaviors, whether the effect is clinically significant and long-term remains in question. Furthermore, because most of these studies were cross-sectional (eg, self-reported online survey), they cannot be used to infer causality and they are vulnerable to bias, which can affect reliability and generalizability of their findings. Such bias includes selection bias (eg, some studies had mostly female or male participants), recall bias (eg, self-reported body weight, BMI, height), and reporting bias (eg, participants may not answer truthfully to questions asked on social and lifestyle behaviors).

### Strengths and limitations

The main strengths of our study were that first, we were able to follow women over time by linked vital statistics and inpatient hospital discharge and ED visit encounter data. Second, though administrative data and birth certificates may have some reliability and validity issues, they provide information on all births at the population level and provide important population-based estimates.

Our study had limitations, including the use and reliability of administrative data and miscoding of BMI classification, gestational weight gain, and race and ethnicity. BMI was based on self-reported prepregnancy weight and height taken from medical records, which can lead to misclassification. Similarly, with gestational weight gain, misclassification could result from BMI misclassification and incorrect report of weight before pregnancy. Self-reported weight tends to be underestimated and individuals who are overweight or obese tend to be more likely to underestimate their weight (22). Pregnant women tend to underreport prepregnancy and delivery weight and overreport gestational weight gain; however, misclassification has been found not to bias the association between BMI, pregnancy weight, and pregnancy outcomes (23). Misclassification of race and ethnicity could have occurred because it was based on information found in administrative data and might not reflect self-reported race and ethnicity. Information was lacking on such factors as diet, physical activity, stress, and neighborhood characteristics, which may be related to obesity and gestational weight gain. Lastly, we excluded pregnant women who had preterm birth from the analysis because early delivery reduces overall gestational weight gain. 

### Conclusion

In South Carolina, the COVID-19 pandemic did not alter trends of gestational weight gain. The pandemic did, however, have a small effect on trends in prepregnancy obesity, with differential effects across racial and ethnic groups. Prepregnancy obesity and gestational weight gain are important public health issues that affect maternal and infant pregnancy outcomes and therefore warrant effective public health interventions. More studies are needed to fully understand the pandemic’s effect on BMI, prepregnancy obesity, and gestational weight gain among women of childbearing age and pregnant women, with an emphasis on racial and ethnic differences. A better understanding of patterns and determinants of pregnancy outcomes after the pandemic can inform effective public health strategies in this population.
